# Sulfur Fumigation-Induced Chemical Transformations in Lily Bulbs (*Lilium brownii* var. *viridulum*): Structural Characterization, Marker Identification, and Toxicity Implications

**DOI:** 10.3390/foods15071228

**Published:** 2026-04-03

**Authors:** Ruiqi Xu, Dingjiang Xuan, Ping Li, Zheng Zhou, Tingyu Zhu, Qi Wu, Lin Zhu, Shuhong Ye, Yan Ding

**Affiliations:** 1 SKL of Marine Food Processing & Safety Control, National Engineering Research Center of Seafood, School of Food Science and Technology, Dalian Polytechnic University, Dalian 116034, China; xuruiqire@163.com (R.X.); drmkmto2@gmail.com (D.X.); liping547464x@163.com (P.L.); zhouzheng971201@163.com (Z.Z.); 15890736616@163.com (T.Z.); wishzzl@163.com (L.Z.); hlcyeshuhong@hotmail.com (S.Y.); 2 Sub-Institute of Agriculture and Food Standardization, China National Institute of Standardization, Beijing 100088, China; wuqi@cnls.ac.cn

**Keywords:** lily bulbs, sulfur fumigation, UPLC-Q-TOF-MS/MS, multivariate statistical analysis

## Abstract

Sulfur fumigation, as a highly effective method for preservation and appearance enhancement, has been widely applied in fruits, vegetables, and food products. However, excessive sulfur fumigation can pose safety risks. Currently, there is limited research on the bound sulfites produced by sulfur fumigation, and no consensus has been reached regarding their structure and toxicity. Using ultra-performance liquid chromatography–quadrupole time-of-flight tandem mass spectrometry (UPLC-Q-TOF-MS/MS), a total of 34 compounds were identified in 12 lily bulb samples subjected to different sulfur fumigation durations. These derivatives were all hypothesized to form via nucleophilic addition to carbon–carbon double bonds. Based on multivariate statistical analysis, 9 characteristic markers were established to rapidly differentiate between non-fumigated (NF) and sulfur-fumigated (SF) samples. The practicality of this strategy was validated using 18 commercial batches. Molecular docking simulations predicted that the modifications might enhance toxicity toward liver injury-related targets, both by altering the spatial conformation of the compounds and because the sulfonic acid group itself serves as an ideal hydrogen-bond acceptor. Overall, mild fumigation led to a gradual accumulation of free sulfur dioxide in lily bulbs, increased the total content of phenolic components and antioxidant capacity, and did not generate excessive bound sulfur dioxide. However, with further extension of fumigation time, the content of sulfur-containing derivatives rose rapidly, accompanied by a noticeable decline in antioxidant activity. This study elucidates the sulfur-driven chemical transformation mechanisms in lily bulbs and establishes a targeted methodology for the quality control and safety assessment of processed herbal products.

## 1. Introduction

The bulb of the lily (*Liliaceae lilium*. L.) is widely recognized as an important edible plant and a source of bioactive pharmaceuticals [1]. As one of the major edible lily varieties in China, lily cultivation holds significant economic and social value, serving as a crucial cash crop that promotes rural economic development and agricultural sustainability, with an annual output exceeding 1,000,000 tons. The lily bulb, as the primary edible and medicinal part, is rich in various bioactive components including phenols, flavonoids, phenolic acids, sterols, steroidal saponins, and steroidal alkaloids [2]. In traditional oriental medicine, lily has been used for moistening the lungs, alleviating cough, and calming the spirit; modern pharmacological studies have demonstrated its therapeutic potential in antioxidant, antitumor, and immunomodulatory activities [3,4,5]. However, during postharvest transportation and storage, the lily bulb is highly susceptible to pest infestation and decay [6], research indicates that room-temperature storage triggers visible brown spotting in lily within six days, progressing to extensive surface browning by day 12 that compromises commercial viability [7]. In addition to typical browning, purple-red discoloration appears on lily bulb scales during storage [8], while fresh-cut lily slices exhibit a decay rate of up to 23.44% after 40 days of storage [9].

To extend the shelf life of lily bulbs and reduce losses, sulfur fumigation, a traditional postharvest treatment, has been widely adopted [10]. This method involves burning sulfur in a closed environment, where sulfur dioxide chemically reacts with water molecules in the lily bulb to form sulfur-containing derivatives [11,12], thereby achieving insecticidal, antimicrobial, and preservative effects. Furthermore, the sulfur dioxide released during sulfur fumigation effectively inhibits polyphenol oxidase activity, blocks the enzymatic browning reaction, and prevents browning of bulb scales during postharvest storage [13]. On the other hand, it exerts a bleaching effect, maintaining the bright and pristine white appearance of lily bulb scales [14]. Although sulfur fumigation has to some extent addressed the issue of preserving lily bulbs, the sulfur-containing derivatives formed may adversely affect their bioactivity [15]. An increasing number of studies have highlighted potential issues associated with sulfur fumigation. Residual sulfur dioxide not only imparts an unpleasant odor but can also cause respiratory problems such as coughing, chest tightness, and throat irritation upon prolonged exposure [16,17]. Moreover, sulfur dioxide and sulfites generated during sulfur fumigation can chemically interact with the intrinsic components of food [18], thereby affecting their composition and quality. For example, in ginseng treated with sulfur fumigation, sulfur dioxide reacts with ginsenosides through esterification to form sulfur-containing derivatives, which have been shown to cause kidney damage in mice [19,20]. In ginger, sulfur fumigation induces an addition reaction of 6-shogaol to form 6-gingesulfonic acid, significantly reducing the anti-cancer and antioxidant properties of ginger [5]. Sulfite treatment can also markedly alter the chemical composition and content of wood ear mushrooms, bean sprouts, and daylily [21,22,23]. Therefore, it is of great significance to investigate the pathways of sulfur-containing derivative formation in sulfur-fumigated lily bulbs. Understanding these pathways is crucial for developing strategies to minimize their formation and ensure the safety of food supplies.

With growing concerns over food and drug safety, a series of advanced analytical methods have been developed in recent years [24]. However, given the complex chemical composition of lily bulbs and the generation of unknown compounds following sulfur fumigation, the application of chromatography-mass spectrometry techniques is particularly important. Ultra-performance liquid chromatography coupled with quadrupole time-of-flight mass spectrometry (UPLC-Q-TOF-MS/MS) has been widely used to separate complex peaks and identify components, thereby characterizing the chemical profiles of food [25,26]. Moreover, the integration of multivariate statistical analysis not only aids in assessing overall differences among samples but also helps identify potential characteristic components [19,27].

Therefore, this study employed UPLC-Q-TOF-MS/MS analysis combined with multivariate statistical analysis to comprehensively characterize the chemical profile of sulfur-fumigated lily bulbs. Specifically, we aimed to identify and elucidate the structures of specific bound sulfur-containing derivatives formed during the fumigation process, predict the formation pathways of these sulfur-containing derivatives through molecular docking analysis and assess their potential nephrotoxicity. By determining free sulfur dioxide content and evaluating antioxidant activity, we assessed the impact of sulfur fumigation on the chemical composition of lily bulbs from multiple dimensions and established reliable sulfur fumigation markers for the quality control and authentication of commercial lily bulb products.

## 2. Materials and Methods

### 2.1. Chemical Reagents and Materials

Acetonitrile was chromatographic grade and purchased from Concord Technology (Tianjin, China). Ultra-pure water was processed by the Milli-Q water purification system (Millipore, Bedford, MA, USA). Formic acid was obtained from Sigma-Aldrich (Darmstadt, Germany). Sulfur sublimed, potassium persulfate, potassium ferricyanide, trichloroacetic acid, and ferric chloride were obtained from Damao (Tianjin, China). 2,2′-Azinobis-(3-ethylbenzothiazoline-6-sulphonate (ABTS), 1,1-diphenyl-2-picryl-hydrazyl radical (DPPH), ascorbic acid, and Trolox were obtained from Aladdin^®^ (Shanghai, China).

The fresh lily samples collected in this experiment were obtained from Lanzhou, Gansu, the origin of Lanzhou lilies. Eighteen batches of commercial lily samples were purchased from shopping malls and online stores in different regions of China. During transport, the product is kept fresh through a low-temperature cold chain throughout the entire journey. According to the regulations of the Pharmacopoeia of the People’s Republic of China (2020 Edition), Associate Professor Yan Ding used morphological and histological methods to identify that the lily samples in this experiment were the bulbs of the lily plant from the family Liliaceae.

### 2.2. Sample Preparation

Sample preparation was conducted using a method previously developed in our laboratory [12,19]. For each replicate, 20 g of fresh lily bulbs were weighed, thoroughly rinsed, and subsequently chopped into small fragments. Each group of samples was prepared three times, and each was processed according to the same procedure. Sulfur-fumigated samples (SF) were prepared using a self-made container. According to the mass ratio of 100:1 (lily bulb slices to sulfur powder), the slices were placed in the upper layer of the container, while sulfur powder was positioned in the lower layer, followed by heating until ignition occurred. The reactor was kept closed to allow for the slow release of SO_2_, which filled the entire container. Subsequently, sulfured lily bulbs from all time groups were dried at 40 °C for 12 h, following the same procedure as the non-fumigated samples (NF). The dried samples were ground into a fine powder using a mortar and passed through a sieve.

An accurately weighed 1.0000 g quantity of the sample powder was mixed with 10 mL of 70% (*v*/*v*) methanol solution and allowed to soak and homogenize. The mixture was subjected to ultrasonic extraction at 40 kHz for 30 min, followed by filtration to collect the supernatant. This ultrasonic extraction process was repeated three times, and the supernatants from all three extractions were combined. The combined extract was filtered using a vacuum filter and then passed through a 0.22 μm filter for HPLC analysis.

### 2.3. Fingerprint Mapping Analysis

#### 2.3.1. HPLC Analysis

The liquid chromatographic conditions of lily bulbs were optimized by using AC Chrom S6000 liquid chromatography and a C_18_ column. After the optimization of the liquid chromatographic conditions such as mobile phase, detection wavelength, and elution gradient, the HPLC conditions were determined as follows: Column: C_18_ column (250 mm × 4.6 mm, 5 μm). Mobile phase: acetonitrile (A)-0.1% (*v*/*v*) formic acid water (B). The elution gradient: 0–20 min, 15–20% A; 20–30 min, 20–25% A; 30–40 min, 25–100% A; 40–45 min, 100% A. Column temperature: 30 °C; flow rate: 1.0 mL/min; injection volume: 10 μL; detection wavelength: 320 nm.

#### 2.3.2. UPLC-Q-TOF-MS/MS Analysis

##### Ultra-Performance Liquid Chromatography

The column used was the ACQUITY UPLC HSS C_18_ column (100 mm × 2.1 mm, 1.8 μm) (Waters Corp., Milford, CT, USA). Column temperature box: 25 °C. Mobile phases: acetonitrile (A) and 0.1% (*v*/*v*) formic acid water (B). The elution gradient: 0–2 min (5–10% A); 2–20 min (10–90% A); 20–22 min (90–100% A). Flow rate: 0.3 mL min^−1^, injection volume: 2 μL.

##### Mass Spectrometry

The Agilent 6550 UPLC-UHD-Q-TOF/MS system (Agilent Technologies, Santa Clara, CA, USA) was used for mass spectrometry detection, which was equipped with an electrospray ionization (ESI) source operating in positive and negative modes, a capillary voltage of 5000 V, a nozzle voltage of 500 V, and a fragmentation voltage of 380 V. The nitrogen flow rate was set at 11 L·min^−1^ at 300 °C, the mass spectrometer dry gas flow was set at 15 L·min^−1^ at 250 °C, and the atomization pressure was 20 psi. The collision induced dissociation capability was set to 30 V in order to obtain fragment ion information. The scanning range was 100–1500 Da, and the dynamic range enhancement was applied to MS test to ensure accurate quality measurement in a wide dynamic range.

The UPLC-Q-TOF-MS/MS data were pre-processed by Masshunter B.06.00 software. The compounds were identified by comparing the chromatographic behavior, empirical molecular formulas, and fragment ions with that of reference compounds or published known compounds in the database.

### 2.4. Determination of Sulfur Dioxide Residue

The free sulfur dioxide residue in lily samples was determined according to the acid-base titration method described in the Chinese National Food Safety Standard GB 5009.34-2022 [28], with slight modifications. Briefly, 5.0 g of lily bulb powder (accurately weighed to 0.0001 g) was placed into a 500 mL round-bottom flask containing 300 mL of ultra-pure water, connected to a distillation apparatus. The receiving flask contained 25 mL of lead acetate solution (20 g/L), with the condenser outlet immersed in the absorption solution. Then, 10 mL of hydrochloric acid (1 + 1, *v*/*v*) was added to the flask via a funnel, and the mixture was heated to distill. The distillation was stopped when approximately 200 mL of distillate was collected. Subsequently, 10 mL of hydrochloric acid (1 + 1, *v*/*v*) was added to the distillate, which was then titrated with an iodine standard solution (0.01 mol/L) using starch solution as an indicator. The titration endpoint was reached when a stable blue color persisted for at least 30 s. A blank test was performed simultaneously. The sulfur dioxide residue (mg/kg) was calculated using the following formula:

*X* = [(*V* − *V*_0_) × *C* × 0.032 × 1000]/*m*

where the following definitions apply:

*X*: the sulfur dioxide content in the sample (mg/kg);

*V*: the volume of iodine standard solution consumed in the sample titration (mL);

*V*_0_: the volume of iodine standard solution consumed in the blank titration (mL);

*C*: the concentration of the iodine standard solution (mol/L);

0.032: the mass of sulfur dioxide (g) equivalent to 1.00 mL of iodine standard solution (1.000 mol/L); *m*: the mass of the sample (g).

### 2.5. Multivariate Statistical Analysis

The correct peak intensity data for each t_R_-m/z pair for the entire batch of samples was compiled in tabular form. The ions from the different samples were considered to be identical as they demonstrated the same t_R_ (tolerance of 0.3 min) and *m*/*z* value. Use SMICA software (SIMCA 15, Umetrics Inc., San Jose, CA, USA.) to complete multivariate statistical analysis, including principal component analysis (PCA), orthogonal partial least squares-discriminant analysis (OPLS-DA), S-Plot, and variable importance for the projection (VIP).

### 2.6. Determination of Antioxidant Activity

#### 2.6.1. DPPH Scavenging

The DPPH free radical scavenging ability was assessed according to the method of Tang with some modifications [4], and Trolox was used as the standard. Take vitamin C solution of different concentrations as positive controls (0.01 mM, 0.1 mM, 1 mM, and 10 mM), and draw a standard curve with Trolox standard solution (y = −0.1278x + 0.3205, R^2^ = 0.9927), and the results were expressed as µg Trolox equivalent per g of dry weight (TE µg/g DW).

#### 2.6.2. ABTS Radical Scavenging

The ABTS radical scavenging assay was based on the method of Yuan, with some modifications [29]. Take vitamin C solution of different concentrations as positive control (0.01 mM, 0.1 mM, 1 mM, and 10 mM), and draw a standard curve with Trolox standard solution (y = −0.2033x + 0.5178, R^2^ = 0.9925). Results are displayed in TE µg/g DW.

#### 2.6.3. Ferric Ion Reducing Antioxidant Power

The ferric ion reducing antioxidant power was assessed according to the method of Zhang, with some modifications [30]. Take vitamin C solution of different concentrations as positive control (0.01 mM, 0.1 mM, 1 mM, and 10 mM), and draw a standard curve with Trolox standard solution (y = 0.0938x + 0.1143, R^2^ = 0.9943). Results are displayed in TE μg/g DW.

### 2.7. Molecular Docking

The molecular docking procedure was performed using AutoDock 4.2 (The Scripps Research Institute, La Jolla, CA, USA). The workflow was as follows: The small molecule structure was optimized using the GAFF force field within the OpenMM molecular dynamics toolkit [31]. The protein model was retrieved from RCSB PDB (https://www.rcsb.org/ accessed on 1 December 2025). Using AutoDock Tools 1.5.6, the protein was designated as the receptor and the flavor compound as the ligand. Structural optimizations including dehydration, hydrogen addition, and charge assignment were performed, and the final structures were converted to the PDBQT format. Grid points and docking dimensions were set to position the grid box to cover the entire protein surface. Subsequently, semi-flexible molecular docking was performed, and the results were saved in PDBQT format. Three-dimensional visualization and analysis were conducted using PyMOL3.1 (Schrödinger, LLC, New York, NY, USA) (https://www.pymol.org accessed on 1 December 2025).

## 3. Results and Discussion

### 3.1. Comparison of Chemical Fingerprint of NF and SF Lily Bulbs

#### 3.1.1. HPLC Analysis

**Figure 1:**
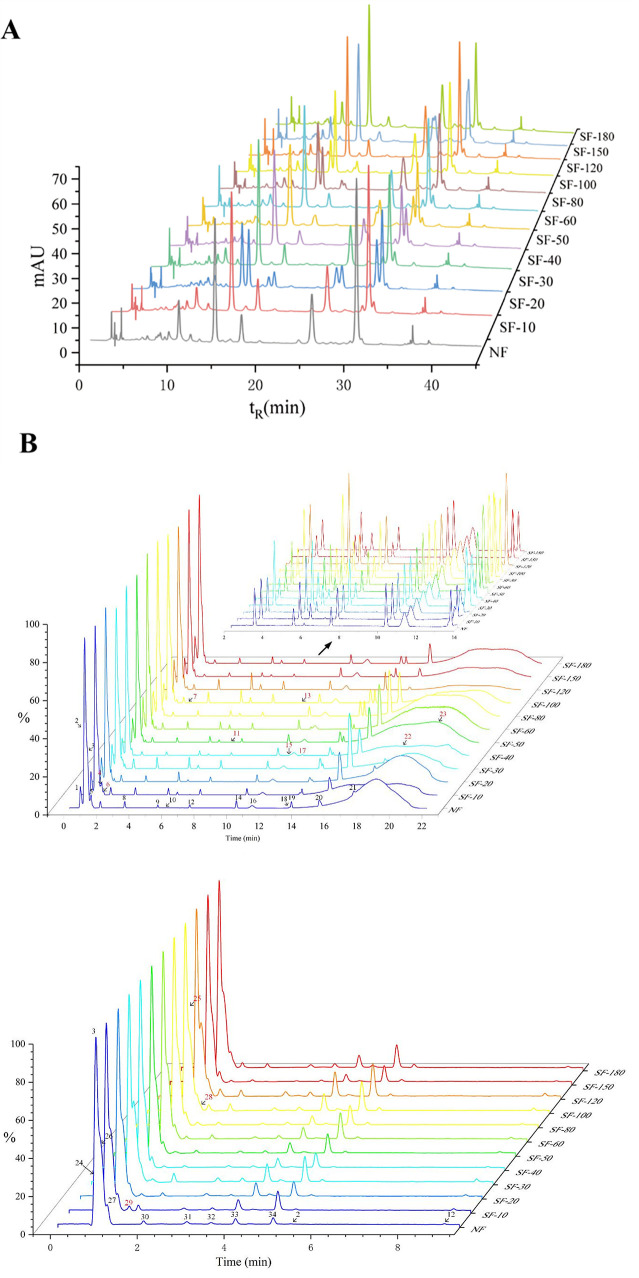
Chromatogram of chemical changes in lily bulbs induced by sulfur fumigation. (**A**): HPLC Chromatograms. (**B**): Total ion chromatograms of NF and SF lily samples.

HPLC was employed to analyze and compare the chemical components of non-fumigated (NF) and sulfur-fumigated (SF) lily bulb samples (Figure 1A). The chromatograms revealed that sulfur fumigation induced significant and time-dependent transformations in the chemical composition. In the control sample (NF), a distinct and sharp main peak was observed at a retention time of approximately 11.2 min. With the extension of sulfur fumigation time (SF-10 to SF-30), the peak area of this compound decreased sharply, indicating rapid consumption of the primary substrate. This finding is consistent with previous studies reporting a significant reduction in the content of major bioactive components in lily bulbs following sulfur fumigation [32]. Concurrent with the decline of primary components, the formation of new compounds became evident. New chromatographic peaks appeared at retention times of approximately 30 min and 50 min. Notably, a peak at approximately 33 min, first detected in the SF-10 sample, exhibited a steady, nearly linear increase in peak area with prolonged treatment time (from SF-10 to SF-180), ultimately becoming the dominant peak in the SF-180 sample. Research indicates that these transformation products are identified as phenylpropenoic acid and furostanol saponin derivatives, formed through nucleophilic addition reactions with sulfur dioxide [33]. The near-linear accumulation of the peak at 33 min likely represents one of these major sulfonated transformation products. The dynamic nature of these chemical reactions was further corroborated by the behavior of intermediates and by-products. For instance, the peak area of the chromatographic peak at approximately 17.8 min initially increased during the SF-10 to SF-40 stages, subsequently diminishing in samples subjected to longer treatment durations characterized by higher peak areas. These findings demonstrate that sulfur fumigation significantly alters the chemical composition of lily bulbs, leading to the degradation or conversion of certain pre-existing compounds and the generation of new ones [34].

#### 3.1.2. UPLC-Q-TOF-MS/MS Analysis

**Figure 2:**
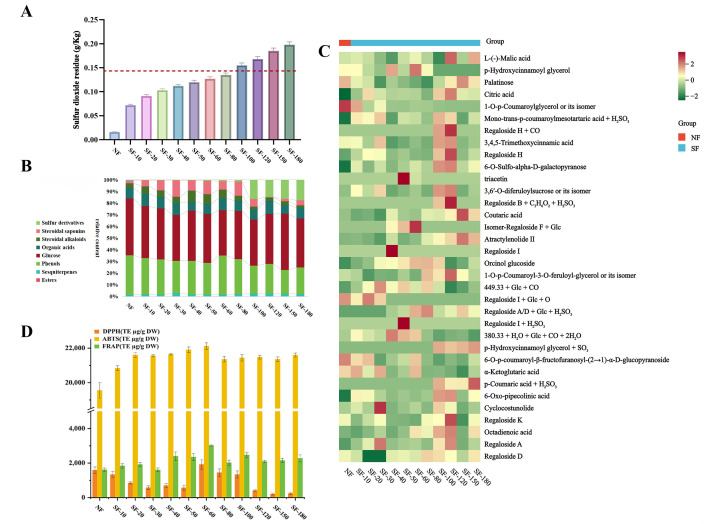
Comprehensive analysis of chemical changes in lily bulbs induced by sulfur fumigation. (**A**): Sulfur Dioxide Residue. (**B**): Chemical Composition Distribution. (**C**): Metabolite Content Heatmap. (**D**): Changes in the Antioxidant Activity of Lily Bulbs.

Following the identification of chemical composition alterations in fresh lily bulbs due to sulfur fumigation treatment using HPLC analysis, this study further employed UPLC-QTOF-MS technology to conduct a comparative analysis of the chemical profiles between non-sulfur fumigated lily samples (NF) and eleven sulfur-fumigated lily samples (SF) at various time intervals (SF-10, SF-20, SF-30, SF-40, SF-50, SF-60, SF-80, SF-100, SF-120, SF-150, and SF-180). The chromatographic data, acquired under both positive and negative ionization modes, enabled the precise mass determination of deprotonated molecular ions, which were then correlated with theoretical mass values to deduce empirical molecular formulas. These formulas were cross-referenced with those of known compounds from extant literature and further validated by examining pseudo-molecular ions and fragment ions, as previously described [20,35]. As shown in Table 1 and Figure 1B, of the 34 compounds identified, which included esters, sesquiterpenes, phenols, polysaccharides, organic acids, steroidal alkaloids, steroidal saponins, and sulfur derivatives, phenols exhibited the greatest diversity, with 12 distinct species. In contrast, only 22 compounds were detected in the NF lily sample. This finding underscores that sulfur fumigation alters the chemical composition of lily samples. Notably, in the SF-10 sample, three novel chromatographic peaks were identified: 4 (citric acid), 6 (mono-trans-*p*-coumaroylmesotartaric acid + H_2_SO_3_), and 29 (6-Oxo-pipecolinic acid), suggesting that sulfur fumigation exerts a substantial impact on lily chemistry from the outset, involving complex initial reactions. With increasing fumigation duration, the influence on sample composition waned but still resulted in the formation of numerous new compounds. In the SF-40 sample, three additional peaks were detected: 15 (isomer-regaloside F + Glc), 17 (regaloside I), and 22 (regaloside A/D + Glc + H_2_SO_3_), while in the SF-50 sample, two new peaks were identified: 11 (triacetin) and 23 (regaloside I + H_2_SO_3_). Importantly, when sulfur fumigation exceeded 100 min, as observed in the SF-100 sample, four new peaks were noted: 7 (regaloside H + CO), 13 (regaloside B + C_3_H_6_O_2_ + H_2_SO_3_), 25 (*p*-hydroxycinnamoyl glycerol + SO_3_), and 28 (*p*-coumaric acid + H_2_SO_3_). Furthermore, peaks 2 (*p*-hydroxycinnamoyl glycerol), 5 (1-O-*p*-coumaroylglycerol or its isomer), and 21 (regaloside I + Glc + O) disappeared after sulfur fumigation, with most peak areas exhibiting significant alterations throughout the fumigation process. These observations suggest that sulfur fumigation markedly alters both the quantity and composition of chemical constituents in lilies, with intricate chemical changes potentially occurring during the fumigation process. The fumigation not only affects the overall chemical content but also modulates the composition, involving rapid reactions in the initial stages and the continuous emergence of new compounds as fumigation time extends. Additionally, as a comparative reference, the free sulfur dioxide content was measured (Figure 2A). Results showed that the residual level exceeded relevant standards only after more than 100 min of sulfur fumigation. From the results, mild sulfur fumigation does not lead to a direct and rapid accumulation of sulfur dioxide residues in lily bulbs. Further analysis regarding the impact on various compounds in lilies will be presented in subsequent chapters.

### 3.2. Effects of Sulfur Fumigation on the Chemical Constituents of Lily Bulbs

Among the eight types of chemical components detected, carbohydrate compounds consistently exhibited the highest content, predominantly sucrose, which is associated with the abundant starch reserves in lily bulbs (Figure 2B). Meanwhile, phenolic compounds were present at relatively high levels in all samples. Overall, as sulfur fumigation time increased, the content of phenolic compounds and their sulfur-containing derivatives showed an upward trend. The primary reason is that sulfur dioxide acts as an efficient inhibitor of polyphenol oxidase, preventing phenolic compounds from being further oxidized into quinones, which also accounts for its whitening effect.

To analyze the spatial distribution of these temporal patterns and evaluate compound-specific dynamic changes, we performed a visualized integrative analysis of the relative metabolite contents in each sample (Figure 2C). The heatmap illustrating compound content variations indicated that sulfur fumigation induced metabolic differences in lily bulbs. Phenolic compounds such as *p*-hydroxycinnamoyl glycerol and regalosides were present at high concentrations in some samples, but their levels significantly decreased with increasing sulfur fumigation intensity. This reduction may be attributed to reactions between phenolic compounds and sulfites during fumigation, forming sulfur-containing derivatives, and to thermal degradation of phenolic compounds during processing [35,36]. Similarly, polysaccharides and phenolic substances with notable antioxidant activity, such as regaloside H and regaloside I, were abundant in the early stages of treatment (SF-10 to SF-60) but declined markedly as treatment duration extended. This decrease may affect the bioactivity and nutritional value of lily bulbs [37]. As the primary active components in lily bulbs, polysaccharides and phenolic compounds effectively scavenge free radicals, so their reduced content may adversely impact the antioxidant activity of lily bulbs [38].

### 3.3. Effect of Sulfur Fumigation on Antioxidant Activity of Lily Bulbs

Given the convenience and rapidity of in vitro antioxidant activity assays, numerous in vitro methodologies have been extensively employed to evaluate the antioxidant capacity of plant extracts [4]. In this study, the antioxidant activity of lily bulbs following sulfur fumigation was assessed using DPPH, ABTS, and FRAP assays. Standard curves for each assay were constructed using trolox at varying concentrations. The free radical scavenging capacity (DPPH and ABTS) and reducing power (FRAP) of lily bulb extracts at different sulfur fumigation durations were represented in terms of trolox equivalent antioxidant capacity (TE µg/g DW), as depicted in Figure 2D.

The results indicate that the antioxidant activity of lily bulbs varies during the sulfur fumigation process. Different durations of sulfur fumigation result in distinct antioxidant capacities of the lily bulbs. Significant differences in DPPH scavenging capacity were observed among the treatment groups. In the NF group, the DPPH radical scavenging capacity was 1586.14 ± 189.74 µg/g DW. This capacity initially increased and then decreased with extended sulfur fumigation, peaking at the SF-60 group with a value of 1918.69 ± 277.57 µg/g DW, followed by a marked decline to below 233.11 ± 49.23 µg/g DW in the SF-180 group. This suggests that sulfur fumigation significantly affects DPPH radical scavenging capacity, with an optimal timing point observed at 60 min. The ABTS radical scavenging capacity of the lily bulb extracts did not exhibit significant variations, whereas the FRAP reducing power showed significant differences among the treatment groups. In the NF group, the FRAP reducing power was 1612.92 ± 100.02 µg/g DW. Similar to the DPPH assay, the FRAP reducing power initially increased and then decreased with prolonged fumigation, reaching its maximum at the SF-60 group (3016.61 ± 38.47 µg/g DW), and then significantly decreased to 2270.34 ± 200.04 µg/g DW in the SF-180 group. This indicates that sulfur fumigation also significantly impacts FRAP reducing power, with an optimal timing point similarly observed at 60 min. The sulfur fumigation treatment significantly influences the antioxidant activity of lily bulbs, with similar trends observed across different antioxidant activity assays. The enhancement of antioxidant activity in SF-60 lily bulbs corresponds with changes in the relative content of phenolic compounds during sulfur fumigation and may be related to the formation of sulfur-containing derivatives or molecular structure. Further investigation is needed to determine whether sulfur-containing derivatives lead to a decrease in antioxidant activity. Studies have shown that changes in antioxidant activity during sulfur fumigation of lily bulbs are associated with alterations in their chemical composition [39].

### 3.4. Exploration of the Potential Transformation Mechanisms of Sulfur-Containing Derivatives

**Figure 3:**
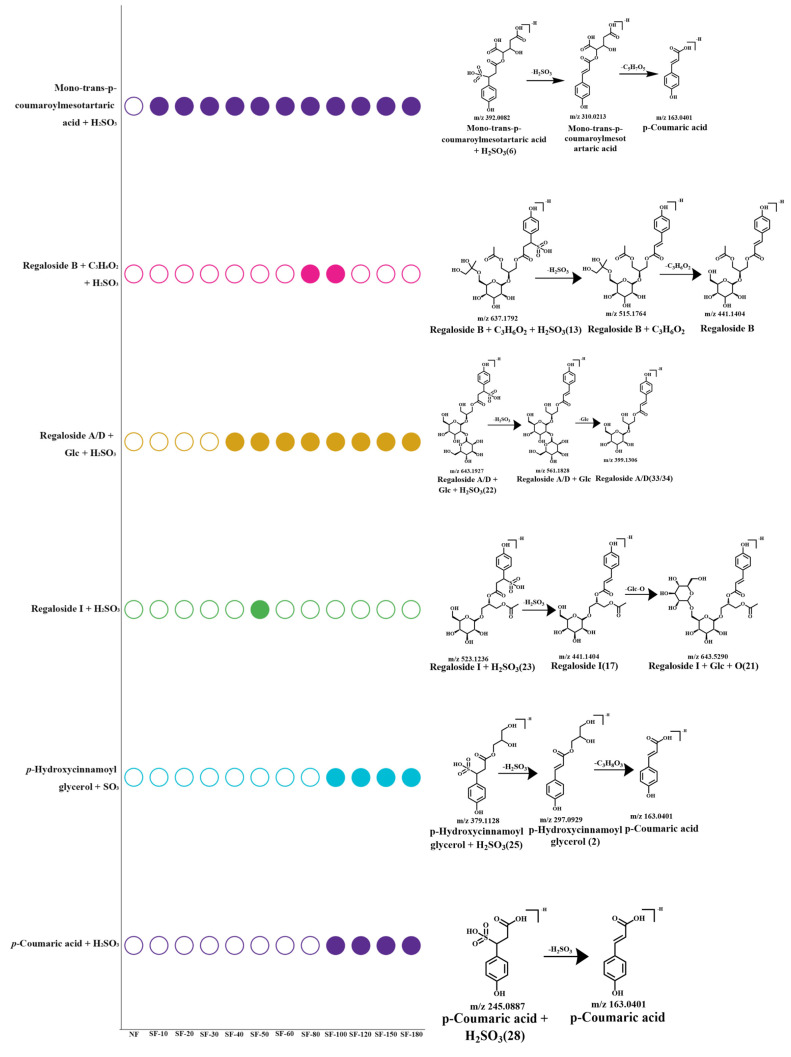
Identification and transformation mechanism analysis of sulfur-containing derivatives in lily bulbs.

The structures and detection times of the six identified sulfur-containing derivatives are presented in Figure 3. All derivatives were detected in negative ion mode, which is likely due to the ease with which the sulfonic acid group loses a proton. Furthermore, it can be inferred that all detected sulfur-containing derivatives were formed by the addition of a sulfonic acid group (-H_2_SO_3_, *m*/*z* 82 Da), and their structures exhibit certain similarities. This allows for a clear determination that the most probable site of addition is the most electron-deficient position within the entire molecule, specifically the carbon atom within the carbon–carbon double bond that is closer to the conjugated group *p*-Coumaric acid (its sulfur-containing derivatives are present in SF-100), as a monophenol, exhibits relatively high stability compared to the previously detected common sulfur-containing derivatives, which are ortho-diphenols, and is less prone to spontaneous oxidation. In contrast, ortho-diphenols are easily oxidized to ortho-quinones, which possess strong electrophilicity. The bisulfite ion in the system acts as a nucleophile and readily undergoes nucleophilic reactions with these quinones. Moreover, even if *p*-Coumaric acid is oxidized to its quinone form, the electron cloud density at its ortho-carbon is still lower than that of the exocyclic carbon–carbon double bond. Furthermore, these derivatives do not possess structures similar to saponins, which might undergo electrophilic addition reactions with sulfurous anhydride via cycloaddition processes. Therefore, it is inferred that the likely site of addition is the exocyclic carbon–carbon double bond. Consequently, the accurate structures of the sulfur-containing derivatives were determined. These sulfur-containing derivatives are all derived from the metabolism of *p*-coumaric acid. A prior study indicated that when *p*-coumaric acid serves as a substrate for enzymatic reactions, the presence of ascorbic acid or catechol leads to the formation of sulfite adducts in the system [40]. Therefore, this phenomenon may be the result of the combined action of the enzyme and the environmental conditions.

The bubble chart illustrates that the reaction product of mono-trans-*p*-coumaroylmesotartaric acid (its sulfur-containing derivatives are present in SF-10) with sulfuric acid had already formed after 10 min of sulfur fumigation, and this compound persisted without degradation as fumigation time extended. When sulfur fumigation exceeded 80 min, an increase in the variety of sulfur-containing derivatives was detected in the samples, and these derivatives continued to remain present without diminishing over time. This pattern suggests that the reactions between sulfur dioxide and compounds during sulfur fumigation may involve irreversible chemical changes, resulting in relatively stable sulfur-containing derivatives [41]. However, the disappearance of certain sulfur-containing derivatives could be attributed to a series of chemical reactions induced by sulfur fumigation, including direct interactions with sulfur dioxide and potential redox processes, which may alter the chemical structures of the derivatives or lead to their decomposition [26]. Notably, the disappearance of sulfur-containing derivatives was only observed in a few samples. For instance, the sulfur-containing compounds of regaloside I and regaloside B were no longer detected after SF-60 and SF-100, while the majority persisted throughout the entire fumigation process. This sustained presence may have significant implications for the safety and functionality of lily bulbs. Simultaneously, we observed that the sulfur-containing derivatives of Regaloside B, *p*-Hydroxycinnamoyl glycerol, and *p*-Coumaric acid appeared after the point at which free sulfur dioxide levels exceeded the permissible standards (SF-100). This may indicate that these compounds could serve as potential markers for indicating excessive sulfur exposure.

### 3.5. Screening and Identification of Lily Bulbs Differentiation Markers

This study encountered challenges in directly analyzing multiple complex mass spectrometry datasets. To address this, multivariate statistical analysis was employed to rapidly identify characteristic components in sulfur-fumigated lilies and evaluate the chemical changes induced by fumigation. Techniques such as box plots, cluster analysis, unsupervised principal component analysis (PCA), and supervised orthogonal partial least squares-discriminant analysis (OPLS-DA) effectively distinguished between non-fumigated (NF) and sulfur-fumigated (SF) lily samples.

**Figure 4:**
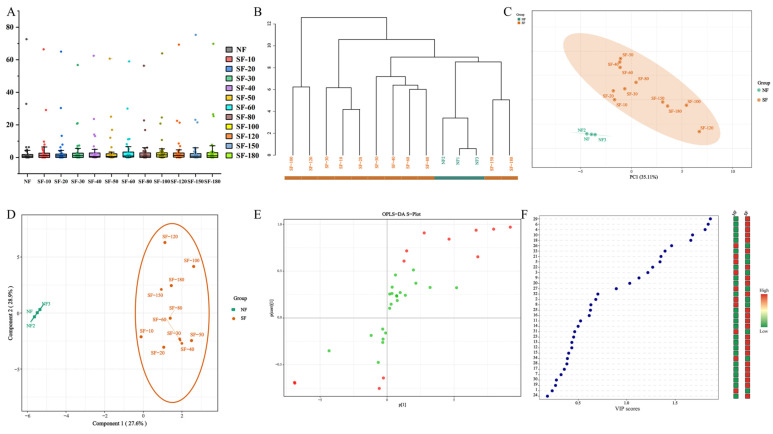
Multivariate statistical analysis of chemical composition in lily bulbs. (**A**): Boxplot. (**B**): Cluster Analysis. (**C**): PCA score plot. (**D**): OPLS-DA score plot. (**E**): S-plot scores. (**F**): VIP value.

Box plots (Figure 4A) showed no significant difference in total metabolite levels between the NF and SF samples (*p* > 0.05). However, cluster analysis (Figure 4B) separated the NF and SF samples into four distinct groups, indicating significant differences in metabolite content within each lily group. PCA clearly segregated NF and SF lilies into two clusters along the first (t [1]) and second (t [2]) principal components. A distinct clustering pattern was also observed between samples fumigated for 10–80 min and those fumigated for 100–180 min (Figure 4C), suggesting a complex reaction process during the initial stage up to 80 min and highlighting significant compositional or content differences between NF and SF lilies. For instance, similar clustering patterns have been reported in trichosanthis, where multivariate statistical analysis clearly differentiated sulfur-fumigated samples from controls along the first and second principal components [42].

OPLS-DA results revealed significant chemical changes in lilies after fumigation, with the initial reaction phase appearing particularly complex (Figure 4D). Based on the OPLS-DA model, an S-plot (Figure 4E) and variable importance in projection (VIP) values (Figure 4F) were used to identify characteristic chemicals. In the S-plot, characteristic chemicals deviated markedly from the origin. Using this plot and a VIP threshold greater than 1, nine characteristic chemicals were identified (peaks **4, 5, 6, 10, 18, 21, 26, 29**, and **33**), including one sulfur-containing derivative.

**Figure 5:**
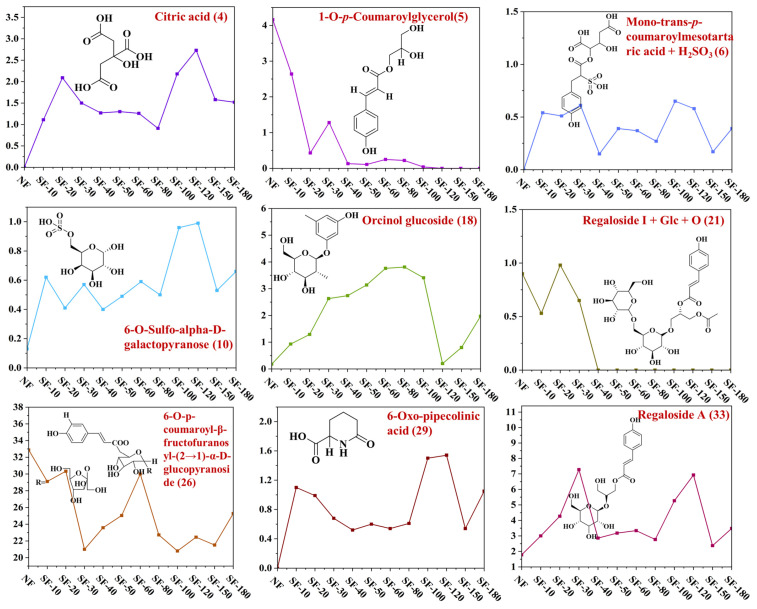
The changes in the marked compounds (**4**, **5**, **6**, **10**, **18**, **21**, **26**, **29**, and **33**).

As shown in Figure 5, structural analysis of these characteristic constituents indicated that the nine identified compounds were predominantly phenolic and sugar derivatives, including one sulfur-containing derivative. Given that lily bulbs contain bioactive components such as regalosides, sulfur fumigation may substantially alter these active constituents, thereby potentially affecting the biological activity of lilies.

Further analysis of the relative content changes in these characteristic chemical components after sulfur fumigation showed the following: A new characteristic compound (No.6) was detected after 10 min of sulfur fumigation, with its concentration ranging from 0.15 to 0.70 mg/mL as the fumigation time increased. This phenomenon aligns with previous studies indicating that sulfur dioxide (SO_2_) can react with endogenous components to form new sulfur-containing derivatives during fumigation [43].The contents of peaks 4 (citric acid), 10 (6-O-sulfo-alpha-D-galactopyranose), 18 (orcinol glucoside), 29 (6-Oxo-pipecolinic acid), and 33 (regaloside A) significantly increased after the start of sulfur fumigation and remained stable with only minor fluctuation during subsequent treatment, without a clear decreasing trend. Specifically, the generation of 6-O-sulfo-alpha-D-galactopyranose further confirms the sulfonation reactions commonly reported in sulfur-fumigated herbs [44]. Furthermore, the increase in organic acids (e.g., citric acid) and certain glycosides may be attributed to the acidic environment induced by SO_2_, which promotes the hydrolysis of complex polysaccharides and larger secondary metabolites [44]. In contrast, the contents of peaks 5 (1-O-*p*-coumaroylglycerol) and 21 (Regaloside I + Glc + O) began to significantly decrease after 40 min of sulfur fumigation and disappeared after 100 min of treatment. Peak 26 (6-O-para-coumaroyl-β-fructofuranosyl-(2→1)-α-D-glucopyranosyl) did not disappear completely, but it showed a downward trend after being exposed to sulfur for 80 min, and eventually reached a certain stable level. According to existing literature, ester bonds and specific glycosidic linkages in coumaroyl derivatives are highly susceptible to degradation or chemical transformation under the acidic and thermal conditions provided by sulfur fumigation [45]. The most significant changes in all characteristic compounds occurred between 30 and 80 min of sulfur fumigation. These observations indicate that the chemical composition of lilies is rapidly affected in the initial stage of sulfur fumigation, after which the impact gradually diminishes. This rapid initial reaction followed by a plateau is consistent with the kinetics of SO_2_ penetration and reaction, where surface reactions occur swiftly before the depletion of reactive substrates or moisture limits further chemical transformations [46].

### 3.6. Quality Evaluation of Commercial Lily Bulbs Samples

Based on the constructed analytical method and the identification of nine characteristic chemical components, the quality of 18 different commercial lily samples was assessed. Through this method, sulfur-containing derivatives of mono-trans-*p*-coumaroylmesotartaric acid + H_2_SO_3_ (6) were detected in 13 commercial lily bulbs (Table 2). The analysis results indicated that all commercial lily samples, except for HN-4, JX-1, JX-2, GS-2, and ZJ-1, were likely subject to sulfur fumigation treatment. This implies that over 70% of the purchased commercial lilies have undergone sulfur fumigation.

### 3.7. Prediction of Kidney Toxicity of Sulfur-Containing Derivatives

**Figure 6:**
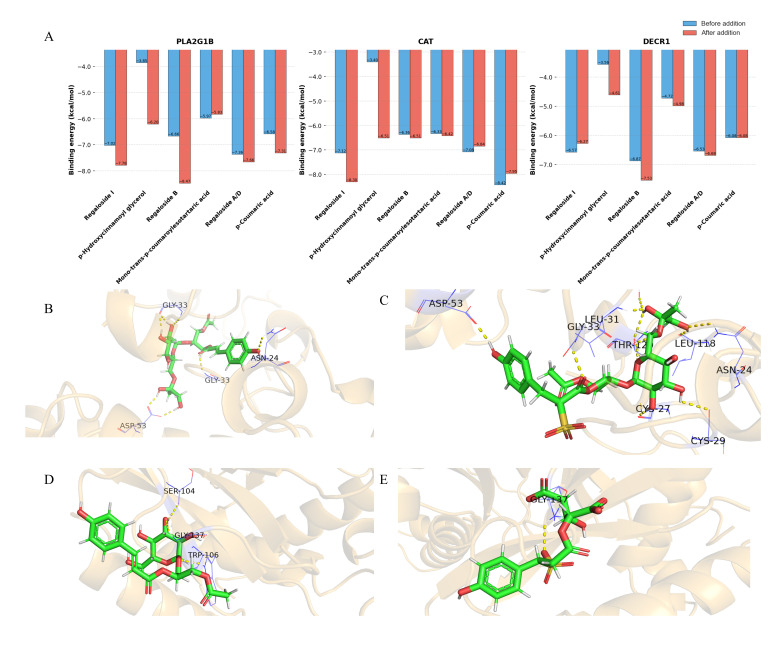
The results and visualization of molecular docking results. (**A**): The binding energy of molecular docking between sulfur-containing derivatives and kidney injury targets (before and after binding). (**B**): Regaloside B and DECR1. (**C**): Regaloside B + H_2_SO_3_ and DECR1. (**D**): Regalosid0e I and CAT. (**E**): Regaloside I + H_2_SO_3_ and CAT.

Considering that the currently reported greatest potential hazard of sulfur fumigation is that some sulfur-containing derivatives may cause damage to the liver and kidneys [47], molecular docking was employed to simulate the interactions between the detected sulfur-containing derivatives, their original metabolites, and kidney injury-related targets (Figure 6A). The results indicated that sulfite adduct formation may lead to a significant reduction in the docking binding energies of Regaloside I (SF-50), *p*-Hydroxycinnamoyl glycerol (SF-100 to SF-180), and Regaloside B (SF-80 to SF-100) with their associated targets, thereby enhancing the binding potential of these compounds to kidney injury-related targets. In contrast, the binding energies of the other three molecules did not change significantly. All sulfur-containing derivatives exhibited low binding energies, suggesting a strong potential binding affinity between these molecules and the targets, which may be one of the reasons for their enhanced hepatorenal toxicity. Therefore, if the sulfur fumigation lasts for more than 40 min, it may result in the formation of more toxic sulfur-containing derivatives.

Visualization results revealed that the primary interactions between these compounds and proteins are hydrogen bonds formed between hydroxyl groups and amino acid residues (Figure 6B). The addition of sulfite did not alter this dominant mode of interaction but rather influenced the molecular-protein interactions primarily by significantly modifying the spatial conformation of the molecules. Additionally, it was observed that the sulfonic acid groups may also form strong hydrogen bonds with amino acid residues. The primary reason lies in the fact that the oxygen atoms of the sulfonic acid group possess lone pair electrons. Especially under neutral conditions, the oxygen may become deprotonated and carry a negative charge, with its extremely high electron cloud density making it an ideal hydrogen bond acceptor. It should be noted that these toxicity assessments are based solely on in silico molecular docking analyses rather than experimental biological or animal data, and thus reflect potential molecular interaction risks rather than confirmed toxicological outcomes.

The fumigation conditions used in this study (including controlled sulfur dioxide concentration, ambient temperature, and relatively low humidity levels) were designed to simulate moderate industrial fumigation practices. Under such conditions, sulfur dioxide may also play a dual role in plant materials. At relatively low concentrations, SO_2_ can act as a signaling molecule or mild abiotic stress factor, potentially inducing antioxidant defense systems, enhancing the activity of enzymes such as superoxide dismutase and catalase, and promoting the accumulation of polyphenolic compounds. This hormesis-like effect has been reported in plant physiology, where low-level sulfur exposure may enhance stress resistance and secondary metabolite biosynthesis.

Based on the preceding analyses, mild sulfur fumigation appears to promote the accumulation of polyphenolic compounds without leading to excessive sulfur dioxide residues or generating a significant amount of sulfur-containing derivatives. Moreover, the two main sulfur-containing derivatives formed showed no significant changes in binding energy in the molecular docking simulations. Taken together, these results suggest that moderate sulfur fumigation, under controlled conditions, may improve product quality while maintaining a relatively low theoretical toxicological risk. However, it must be emphasized that these conclusions are preliminary and based on computational predictions. Given the potential toxicity of sulfur-containing derivatives and the complexity of their detection, further in vitro and in vivo studies are required to comprehensively evaluate the safety of sulfur fumigation.

## 4. Conclusions

In this study, the impact of sulfur fumigation on the chemical composition and antioxidant activity of lily bulbs was systematically evaluated using UPLC-Q-TOF-MS/MS combined with multivariate statistical analysis. A total of 34 compounds were identified in both NF and SF lily bulbs. The results showed that sulfur fumigation significantly altered the content of chemical constituents in lily bulbs, particularly the phenolic compounds. Sulfur fumigation induced the chemical transformation of phenolic compounds, resulting in the formation of six sulfur-containing derivatives: mono-trans-*p*-coumaroylmesotartaric acid + H_2_SO_3_, regaloside B + C_3_H_6_O_2_ + H_2_SO_3_, regaloside D + Glc + H_2_SO_3_, regaloside I + H_2_SO_3_, *p*-hydroxycinnamoyl glycerol + SO_3_, and *p*-coumaric acid + H_2_SO_3_. Based on fragment information, it can be clearly inferred that the formation of these derivatives is primarily attributed to the nucleophilic addition reaction between the electron-deficient carbon–carbon double bonds in the phenolic compounds and sulfite during the sulfur-fumigation process. The molecular docking simulation further indicates that when the sulfur fumigation treatment time exceeds 40 min, the addition of sulfites may alter the toxic effects of the original compound on kidney-related targets. Additionally, although sulfur-fumigation treatment can inhibit the enzyme-catalyzed reactions of polyphenols, prolonged fumigation reduced the antioxidant capacity of lily bulbs. The DPPH, ABTS, and FRAP antioxidant indices all peaked at 60 min of fumigation and subsequently declined. Through multivariate statistical analysis, nine characteristic chemical components were identified, primarily consisting of sugars and phenolic compounds. These components effectively differentiated NF from SF. Based on these markers, 18 batches of commercial lily bulb samples were further assessed. The results indicated that over 70% of the samples had likely undergone sulfur fumigation prior to drying. This finding highlights the widespread use of sulfur fumigation in commercial lily bulb processing and suggests potential risks to the safety and functionality of lily bulbs.

## Figures and Tables

**Table 1:** Chemical component information of 34 labeled peaks in the BPI and TIC spectra.

No.	t_R_ (min)	MS/MS Fragment Ions	*m*/*z* (<10 ppm)	Formula	Identity	Class	Relative Content (%)											
							NF	SF-10	SF-20	SF-30	SF-40	SF-50	SF-60	SF-80	SF-100	SF-120	SF-150	SF-180
1	0.70	115.0037, 89.0246	175.1196	C_4_H_6_O_5_	L-(-)-Malic acid	Organic acids	6.30	6.43	6.20	5.55	4.93	6.23	6.60	5.98	4.64	8.54	5.90	7.74
24	0.86	588.3892	665.2210	C_34_H_55_O_8_N	380.33 + H_2_O + Glc + CO + 2H_2_O	Steroidal alkaloids	6.30	9.67	7.85	7.10	14.04	12.06	11.31	5.63	3.85	4.67	4.93	3.36
2	0.91	163.0413; 145.0288; 119.0403	280.0929	C_12_H_14_O_5_	*p*-Hydroxycinnamoyl glycerol	Phenols	4.33	4.32	2.39	1.22	6.79	2.25	8.18	3.93	-	-	-	-
25	0.93	297.0929; 163.0413; 119.0403	379.1128	C_12_H_14_SO_8_	*p*-Hydroxycinnamoyl glycerol + SO_3_	Sulfur derivatives	-	-	-	-	-	-	-	-	24.49	21.36	23.09	26.49
3	0.97	179.0561	341.1082	C_12_H_22_O_11_	Sucrose	Disaccharide	72.64	66.37	64.97	56.76	62.45	60.65	58.95	56.29	63.87	69.25	75.27	69.76
26	1.05	145.0178	487.1383	C_21_H_28_O_13_	6-O-*p*-coumaroyl-β-fructofuranosyl-(2→1)-α-D-glucopyranoside	Phenols	32.87	29.1	30.33	21	23.58	25.04	30	22.72	20.8	22.45	21.51	25.28
27	1.17	144.9423; 101.0285; 57.0375	145.0164	C_5_H_6_O_5_	α-Ketoglutaric acid	Organic acids	4.78	5.29	3.88	5.28	2.8	3.24	5.11	2.79	2.84	2.63	2.03	3.35
28	1.26	163.0398; 145.0362	245.0887	C_9_H_9_SO_6_	*p*-Coumaric acid + H_2_SO_3_	Sulfur derivatives	-	-	-	-	-	-	-	-	1.29	0.88	0.9	1.85
4	1.30	173.0092, 111.0088	231.0852	C_6_H_8_O_7_	Citric acid	Organic acids	-	1.11	2.09	1.50	1.27	1.30	1.26	0.91	2.18	2.73	1.58	1.52
5	1.41	145.0184	407.1170	C_21_H_20_O_7_	1-O-*p*-Coumaroylglycerol or its isomer	Phenols	4.16	2.64	0.43	1.28	0.131	0.11	0.25	0.22	0.04	-	-	-
29	1.45	-	142.0533	C_6_H_9_NO_3_	6-Oxo-pipecolinic acid	organic acids	-	1.1	0.99	0.68	0.52	0.6	0.54	0.61	1.5	1.54	0.54	1.05
6	1.50	163.0413; 310.0213	392.0082	C_14_H_16_SO_11_	Mono-trans-*p*-coumaroylmesotartaric acid + H_2_SO_3_	Sulfur derivatives	-	0.54	0.51	0.61	0.15	0.39	0.37	0.27	0.65	0.58	0.17	0.39
30	2.00	102.0606	232.1196	C_15_H_20_3	Cyclocostunolide	Sesquiterpenes	0.85	1.17	0.86	2.84	0.57	0.74	0.91	0.62	1.76	1.34	0.48	0.83
7	2.42	399.1296, 381.1191, 235.0819, 163.0402, 145.0296	447.1484	C_19_H_2_4O_11_	Regaloside H + CO	Phenols	-	-	-	-	-	-	-	-	0.05	0.08	-	-
31	3.01	179.0289; 161.0282	415.1186	C_18_H_24_O_11_	Regaloside K	Phenols	0.82	0.57	0.72	0.84	0.55	0.54	0.51	0.83	0.86	1.3	0.4	0.83
8	3.48	153.0496	256.1167	C_12_H_14_O_5_	3,4,5-Trimethoxycinnamic acid	Phenols	1.49	1.51	1.19	1.82	0.84	0.95	1.08	0.97	1.96	2.01	1.04	1.16
32	3.57	91.0424; 59.0125	253.0709	C_8_H_12_O_2_	Octadienoic acid	organic acids	0.53	0.47	0.55	0.65	0.5	0.58	0.9	0.79	1.03	1.13	0.52	0.91
33	4.13	163.0380; 145.2097	399.1227	C_18_H_24_O_10_	Regaloside A	Phenols	1.78	3	4.27	7.28	2.86	3.18	3.34	2.77	5.27	6.93	2.37	3.48
34	4.47	237.0709; 163.0380; 145.2097	399.1231	C_18_H_24_O_10_	Regaloside D	Phenols	0.53	0.46	-	-	0.5	0.52	0.48	0.68	0.65	0.57	0.4	0.63
9	5.48	237.0767, 219.0658, 163.0398, 145.0294	423.1283	C_18_H_24_O_10_	Regaloside H	Phenols	0.70	1.32	1.84	1.76	0.99	1.14	0.92	0.87	2.29	3.17	0.99	1.39
10	6.04	-	261.0746	C_6_H_12_SO_9_	6-O-Sulfo-alpha-D-galactopyranose	Polysaccharides	0.13	0.62	0.41	0.57	0.40	0.49	0.59	0.50	0.96	0.99	0.53	0.66
11	6.83	181.0412, 241.0687	241.0683	C_9_H_14_O_6_	triacetin	Esters	-	-	-	-	-	0.21	0.02	-	-	-	-	-
12	7.40	517.1565, 499.1458, 337.0926, 265.0822, 193.0505, 175.0399	717.2031	C_32_H_38_O_17_	3,6′-O-diferuloylsucrose or its isomer	Phenolic	0.82	1.25	0.99	1.41	0.67	0.77	0.88	0.72	1.65	1.73	0.72	0.91
13	9.19	515.1764, 441.1404, 263.0536; 163.0401,	597.1792	C_23_H_32_O_16_S	Regaloside B + C_3_H_6_O_2_ + H_2_SO_3_	Sulfur derivatives	-	-	-	-	-	-	-	-	0.23	0.48	-	-
14	10.29	163.0402, 145.0144	328.3217	C_14_H_14_O_8_	Coutaric acid	Phenols	1.56	1.43	1.46	1.32	1.82	1.61	1.57	1.81	1.79	1.96	2.51	2.05
15	11.03	415.1448, 235.0830, 193.1648	615.5815	C_25_H_36_O_16_	Isomer-Regaloside F + Glc	Phenols	-	-	-	-	0.13	0.20	0.37	-	-	-	-	-
16	11.25	123.1149; 232.8991;	250.1788	C_15_H_20_O_2_	Atractylenolide II	Sesquiterpenes	1.71	1.74	1.65	1.44	2.14	1.64	1.77	2.26	2.53	2.72	3.31	2.90
17	11.79	399.1294, 381.1193, 163.0403, 145.0296	443.2339	C_20_H_26_O_11_	Regaloside I	Phenols	-	-	-	-	0.32	-	-	-	-	-	-	-
18	13.39	230.9120	304.3003	C_13_H_18_O_7_	Orcinol glucoside	Phenols	0.18	0.93	1.29	2.63	2.74	3.14	3.76	3.81	3.41	0.20	0.80	1.97
19	13.71	354.5274, 235.0611, 193.0500, 163.0399	437.1940	C_22_H_22_O_8_	1-O-*p*-Coumaroyl-3-O-feruloyl-glycerol or its isomer	Phenols	1.54	1.35	0.35	0.78	0.73	1.25	0.75	2.82	2.42	3.76	2.19	1.41
20	15.16	611.3785; 449.3279,	641.5130	C_34_H_56_O_11_	449.33 + Glc + CO	Steroidal saponins	3.54	7.90	13.27	20.71	13.05	16.92	11.11	16.79	10.70	0.92	3.61	7.19
21	17.50	163.1470; 398.7905; 235.1387	643.5290	C_26_H_36_O_17_	Regaloside I + Glc + O	Phenols	0.90	0.53	0.98	0.65	-	-	-	-	-	-	-	-
22	17.64	561.1828, 399.1306, 163.0401, 145.0295	643.1927	C_24_H_36_SO_18_	Regaloside A/D + Glc + H_2_SO_3_	Sulfur derivatives	-	-	-	-	0.46	0.61	1.57	1.82	0.64	0.87	1.39	0.95
23	18.29	441.1404, 381.1196, 163.0401,	523.1236	C_20_H_28_SO_14_	Regaloside I + H_2_SO_3_	Sulfur derivatives	-	-	-	-	-	0.14	-	-	-	-	-	-

**Table 2:** Screening for 9 chemical markers in commercial lily bulbs.

No.	Samples	Chemical Markers								
		4	5	18	10	21	29	6 *	26	33
1	HN-1	√	√	-	√	-	√	√	√	√
2	HN-2	√	√	-	√	-	√	√	√	-
3	HN-3	√	√	√	√	-	-	√	√	√
4	HN-4	√	√	-	√	√	√	-	√	-
5	HeB-1	√	√	-	√	-	√	√	√	√
6	HeB-2	√	√	-	√	-	√	√	-	-
7	HeB-3	-	√	√	√	√	√	√	√	√
8	JX-1	√	√	√	√	√	-	-	√	√
9	JX-2	√	√	√	√	√	√	-	√	-
10	GS-1	√	√	√	√	√	√	√	√	√
11	GS-2	√	√	√	√	√	√	-	√	√
12	HB-1	√	√	-	√	-	-	√	√	√
13	HB-2	√	√	√	√	√	√	√	√	√
14	FJ-1	√	√	-	√	√	√	√	-	√
15	FJ-2	-	√	-	√	√	√	√	√	√
16	ZJ-1	√	√	-	√	√	√	-	√	√
17	ZJ-2	-	√	-	√	√	√	√	√	√
18	SC-1	√	√	-	√	√	-	√	√	-

HN: Hunan Province; HeB: Hebei Province; JX: Jiangxi Province; GS: Gansu Province; HB: Hubei Province; FJ: Fujian Province; ZJ: Zhejiang Province; SC: Sichuan Province; √: Detected in the sample; -: Not detected; *: sulfur-containing derivative.

## Data Availability

The original contributions presented in this study are included in the article. Further inquiries can be directed to the corresponding author.

## References

[1] Wang P., Li J., Attia F.A.K., Kang W., Wei J., Liu Z., Li C. (2019). A critical review on chemical constituents and pharmacological effects of Lilium. Food Sci. Hum. Wellness.

[2] Zhou J., An R., Huang X. (2021). Genus Lilium: A review on traditional uses, phytochemistry and pharmacology. J. Ethnopharmacol..

[3] Li S., Bao F., Cui Y. (2021). Immunoregulatory activities of the selenylated polysaccharides of Lilium davidii var. unicolor Salisb in vitro and in vivo. Int. Immunopharmacol..

[4] Tang Y.C., Liu Y.J., He G.R., Cao Y.W., Bi M.M., Song M., Yang P.-P., Xu L.-F., Ming J. (2021). Comprehensive analysis of secondary metabolites in the extracts from different lily bulbs and their antioxidant ability. Antioxidants.

[5] Zhang W.H., Luo H.Y., Fang J., Zhao C.L., Chan K.C., Chan Y.M., Dong C.-X., Chen H.-B., Zhao Z.-Z., Li S.-L. (2022). Impact of sulfur fumigation on ginger: Chemical and biological evidence. J. Agric. Food Chem..

[6] Liang Z.X., Zhang J.Z., Xin C., Li D., Sun M.Y., Shi L. (2022). Analysis of edible characteristics, antioxidant capacities, and phenolic pigment monomers in Lilium bulbs native to China. Food Res. Int..

[7] Ji J., Li X., Guo X., Liu X., Ma X., Wang M., Wang Y., Jin L., Zhou L. (2025). X-ray irradiation delayed the browning development of fresh-cut bulbs of Lanzhou Lily (*Lilium davidii*) during room temperature storage. Radiat. Phys. Chem..

[8] Fan W., Bai P., Chen R., Guo T., Tian Y., Tian H., Ren H. (2024). Postharvest Light Irradiation Induces Anthocyanin Accumulation in Fresh-Cut Lily Bulb (*Lilium davidii* var. *unicolor*) Scales. J. Food Process. Preserv..

[9] Gao Y., Du J., Li Y., Su J., Li X., Jiang Y., Leng C. (2025). Study on the change rules of lily storage quality at different temperatures. Storage Process.

[10] Li X.Y., Xu J.D., Xu J., Kong M., Zhou S.S., Mao Q., Brand E., Chen H.-B., Liu H.-Q., Li S.-L. (2016). UPLC-QTOF-MS based metabolomics coupled with the diagnostic ion exploration strategy for rapidly evaluating sulfur-fumigation caused holistic quality variation in medicinal herbs, Moutan Cortex as an example. Anal. Methods.

[11] Deng A.P., Kang C.Z., Kang L.P., Lyu C.G., Zhang W.J., Wang S., Wang H.-Y., Nan T.-G., Zhou L., Huang L.-Q. (2022). Practical protocol for comprehensively evaluating sulfur-fumigation of Baizhi based on metabolomics, pharmacology, and cytotoxicity. Front. Pharmacol..

[12] Li P., Zhang Y., Ding Y., Wu Q., Liu Z., Zhao P., Zhao G., Ye S. (2022). Discrimination of raw and sulfur-fumigated ginseng based on Fourier transform infrared spectroscopy coupled with chemometrics. Microchem. J..

[13] Guo A.L., Chen L.M., Wang Y.M., Liu X.Q., Zhang Q.W., Gao H.M., Wang Z.-M., Xiao W., Wang Z.Z. (2014). Influence of sulfur fumigation on the chemical constituents and antioxidant activity of buds of Lonicera japonica. Molecules.

[14] Li P., Li J., Ding Y., Wu Q., Chen D., Chen J., Liu Z., Ye S. (2025). Influence of sulfur fumigation on the volatile composition of lily bulbs evaluated by HS-SPME/GC–MS and multivariate statistical analysis. J. Sci. Food Agric..

[15] Kang C., Zhao D., Zhou T., Liu D.H., Lv C., Wang S., Kang L., Yang J., Zhan Z.-L., Huang L. (2017). A practical protocol for comprehensive evaluation of sulfur-fumigation of Gastrodia Rhizoma using metabolome and health risk assessment analysis. J. Hazard. Mater..

[16] Duan S.M., Xu J., Bai Y.J., Ding Y., Kong M., Liu H.H., Li X.-Y., Zhang Q.-S., Chen H.-B., Liu L.-F. (2016). Sulfur dioxide residue in sulfur-fumigated edible herbs: The fewer, the safer?. Food Chem..

[17] Chan K.C., Zhang W.H., Chan Y.M., Li H.L., Fang J., Luo H.Y., Xu J. (2024). Tryptophan sulfonate: A new chemical marker for accurate and efficient inspection of sulfur-treated food products. Food Chem..

[18] Jiang X., Huang L.F., Zheng S.H., Chen S.L. (2013). Sulfur fumigation, a better or worse choice in preservation of Traditional Chinese Medicine?. Phytomedicine.

[19] Zhang Y., Wang B., Zhao P., He F., Xiao W., Zhu J., Ding Y. (2021). A comprehensive evaluation protocol for sulfur fumigation of ginseng using UPLC-Q-TOF-MS/MS and multivariate statistical analysis. LWT.

[20] He J., Jiang J., Xie T., Liu Y., Cai H., Xiao S., Cai Z., Chen T. (2023). Exploring the nephrotoxicity of sulfur-containing derivatives in sulfur-fumigated Panacis Quinquefolii Radix based on chemical profiling and untargeted metabolomics. J. Ethnopharmacol..

[21] Ren Y., Huang J., Wang X., Wang Y., Li H., Yue T., Gao Z. (2022). Effects of sulfite treatment on the quality of black fungus. Food Chem..

[22] Li Y., Zhou Z., Wu Q., Chen B., Ye S., Cui Y., Ding Y. (2024). Untargeted metabolomics combined with vitro antioxidant to comprehensively evaluate the effect of sodium sulfite immersion on the holistic quality of mung bean sprouts. J. Food Sci..

[23] Li J., Zhou Z., Wu Q., Liu Z., Zhu L., Ye S., Ding Y. (2025). Untargeted metabolomics reveals the formation pathways of bound sulfite in sodium metabisulfite-soaked Hemerocallis citrina Baroni. Food Chem..

[24] Liu H., Wang S.Y., Zhu J.H., Kong M., Zhou S.S., Li S.L., Zhu H. (2022). Effects and contributory factors of sulfur-fumigation on the efficacy and safety of medicinal herbs evaluated by meta-analysis. J. Ethnopharmacol..

[25] Sun X., Cui X.B., Wen H.M., Shan C.X., Wang X.Z., Kang A., Chai C., Li W. (2017). Influence of sulfur fumigation on the chemical profiles of Atractylodes macrocephala Koidz. evaluated by UFLC–QTOF–MS combined with multivariate statistical analysis. J. Pharm. Biomed. Anal..

[26] Li Z., Huang J., Wang L., Li D., Chen Y., Xu Y., Li L., Xiao H., Luo Z. (2024). Novel insight into the role of sulfur dioxide in fruits and vegetables: Chemical interactions, biological activity, metabolism, applications, and safety. Crit. Rev. Food Sci. Nutr..

[27] Hou H.D., Wu C.Y., Zhou J., Xu J.D., Long F., Zhu J.H., Zhou S.-S., Zhang W., Mao Q., Shen H. (2022). Holistic quality evaluation of commercial Agastache rugosa by multiple chromatographic and chemometric analysis. J. Pharm. Biomed. Anal..

[28] (2022). National Food Safety Standard - Determination of Sulfur Dioxide in Foods.

[29] Yuan M., Yan Z., Liu Y., Chen D., Yang Z., He L., Zhang Z. (2019). Chemical profiles, antioxidant activity and acute toxicity of raw and sulfur-fumigated Smilacis Glabrae Rhizoma. J. Ethnopharmacol..

[30] Zhang R., Yuen A.K., Magnusson M., Wright J.T., de Nys R., Masters A.F., Maschmeyer T. (2018). A comparative assessment of the activity and structure of phlorotannins from the brown seaweed Carpophyllum flexuosum. Algal Res..

[31] Eastman P., Swails J., Chodera J.D., McGibbon R.T., Zhao Y., Beauchamp K.A., Wang L.-P., Simmonett A.C., Harrigan M.P., Stern C.D. (2017). OpenMM 7: Rapid development of high performance algorithms for molecular dynamics. PLoS Comput. Biol..

[32] Li L., Zhang Z., Cai B. (2006). Effect of sulfur fumigation on effective components of lily. J. Shanghai Univ. Tradit. Chin. Med..

[33] Zhang R., Yang Y., Deng A., Kang L., Cheng M., Kang C., Guo L. (2023). Effect of sulfur fumigation on quality and safety of lily medicinal materials. China J. Chin. Mater. Medica.

[34] Tan C., Chen M., Chen H., Lin Z. (2025). Rapid discrimination of sulfur-fumigated lily by mid-infrared, near-infrared and synchronous fluorescence spectroscopy and chemometrics: A comparative study. Spectrochim. Acta Part A Mol. Biomol. Spectrosc..

[35] Wu X., Hou J., Zhang Z., Chen L., Ni H., Qian Y., Wu W., Long H., Zhang L., Li F. (2022). In-depth exploration and comparison of chemical constituents from two Lilium species through offline two-dimensional liquid chromatography combined with multimode acquisition of high-resolution mass spectrometry. J. Chromatogr. A.

[36] Wang B., Nie C., Li T., Zhao J., Fan M., Li Y., Qian H., Wang L. (2022). Effect of boiling and roasting on phenolic components and their bioaccessibilities of highland barley. Food Res. Int..

[37] Wang M., Tang H.P., Bai Q.X., Yu A.Q., Wang S., Wu L.H., Fu L., Wang Z.-B., Kuang H.X. (2024). Extraction, purification, structural characteristics, biological activities, and applications of polysaccharides from the genus Lilium: A review. Int. J. Biol. Macromol..

[38] Jin L., Zhang Y., Yan L., Guo Y., Niu L. (2012). Phenolic compounds and antioxidant activity of bulb extracts of six Lilium species native to China. Molecules.

[39] Guo T., Feng W.H., Liu X.Q., Gao H.M., Wang Z.M., Gao L.L. (2016). Characterization of the processing of dry lily (*Lilium davidii* Duch.) bulbs by mid-infrared spectroscopy. Anal. Lett..

[40] Markakis P., Embs R.J. (1966). Effect of sulfite and ascorbic acid on mushroom phenol oxidase. J. Food Sci..

[41] Zhang C.L., Liu C., Nie S.R., Zhang Y., Guo J.H., Liu C. (2024). Near-infrared colorimetric fluorescent probe for detecting HSO_3−_ and its practical application. J. Mol. Struct..

[42] Kang C., Lv C., Yang J., Kang L., Ma W., Zhang W., Wang S., Wang T., Sun J., Ge Y. (2020). A practical protocol for a comprehensive evaluation of sulfur fumigation of trichosanthis radix based on both non-targeted and widely targeted metabolomics. Front. Plant Sci..

[43] Xu W., Jin H., Wang Y., Wei F., Liu J. (2025). Sulfur fumigation of botanical drugs: Impact on chemical composition and pharmacological properties, and advances in detection technologies. Front. Pharmacol..

[44] Chan Y.M., Lu B.W., Zhang W.H., Chan K.C., Fang J., Luo H.Y., Du J., Zhao Z.-Z., Chen H.-B., Dong C. (2023). Impact of sulfur fumigation on the chemistry of dioscoreae rhizoma (Chinese yam). ACS Omega.

[45] Makila L., Laaksonen O., Alanne A.L., Kortesniemi M., Kallio H., Yang B. (2016). Stability of hydroxycinnamic acid derivatives, flavonol glycosides, and anthocyanins in black currant juice. J. Agric. Food Chem..

[46] Kan W.L.T., Ma B., Lin G. (2011). Sulfur fumigation processing of traditional Chinese medicinal herbs: Beneficial or detrimental?. Front. Pharmacol..

[47] Chen C.J., Cheng M.C., Hsu C.N., Tain Y.L. (2023). Sulfur-containing amino acids, hydrogen sulfide, and sulfur compounds on kidney health and disease. Metabolites.

